# The effects of prolonged sitting behavior on resting-state brain functional connectivity in college students post-COVID-19 rehabilitation: A study based on fNIRS technology

**DOI:** 10.1016/j.smhs.2024.06.002

**Published:** 2024-06-04

**Authors:** Xiaocong Yan, Ying Qin, Haifeng Yu, Zhenghao Xue, Desheng Jiang, Limin Huang

**Affiliations:** a Graduate School, Harbin Sport University, Harbin, 150008, China; b College of Sports and Human Sciences, Harbin Sport University, Harbin, 150008, China

**Keywords:** fNIRS, COVID-19, Sedentary behavior, Resting state, College students, Functional connectivity

## Abstract

Functional near-infrared spectroscopy (fNIRS) was used to explore the effects of sedentary behavior on the brain functional connectivity characteristics of college students in the resting state after recovering from Corona Virus Disease 2019 (COVID-19). Twenty-two college students with sedentary behavior and 22 college students with sedentary behavior and maintenance of exercise habits were included in the analysis; moreover, 8 ​min fNIRS resting-state data were collected. Based on the concentrations of oxyhemoglobin (HbO_2_) and deoxyhemoglobin (HbR) in the time series, the resting-state functional connection strength of the two groups of subjects, including the prefrontal cortex (PFC) and the lower limb supplementary motor area (LS), as well as the functional activity and functional connections of the primary motor cortex (M1) were calculated. The following findings were demonstrated. (1) Functional connection analysis based on HbO_2_ demonstrated that in the comparison of the mean functional connection strength of homologous regions of interest (ROIs) between the sedentary group and the exercise group, there was no significant difference in the mean functional strength of the ROIs between the two groups (p>0.05). In the comparison of the mean functional connection strengths of the two groups of heterologous ROIs, the functional connection strengths of the right PFC and the right LS (p=0.0097), the left LS (p=0.0127), and the right M1 (p=0.0305) in the sedentary group were significantly greater. The functional connection strength between the left PFC and the right LS (p=0.0312) and the left LS (p=0.0370) was significantly greater. Additionally, the functional connection strength between the right LS and the right M1 (p=0.0370) and the left LS (p=0.0438) was significantly greater. (2) Functional connection analysis based on HbR demonstrated that there was no significant difference in functional connection strength between the sedentary group and the exercise group (p>0.05) or between the sedentary group and the exercise group (p>0.05). Similarly, there was no significant difference in the mean functional connection strength of the homologous and heterologous ROIs of the two groups. Additionally, there was no significant difference in the mean ROIs functional strength between the two groups (p>0.05). Experimental results and graphical analysis based on functional connectivity indicate that in this experiment, college student participants who exhibited sedentary behaviors showed an increase in fNIRS signals. Increase in fNIRS signals among college students exhibiting sedentary behaviors may be linked to their status post-SARS-CoV-2 infection and the sedentary context, potentially contributing to the strengthened functional connectivity in the resting-state cortical brain network. Conversely, the fNIRS signals decreased for the participants with exercise behaviors, who maintained reasonable exercise routines under the same conditions as their sedentary counterparts. The results may suggest that exercise behaviors have the potential to mitigate and reduce the impacts of sedentary behavior on the resting-state cortical brain network.

## List of abbreviations:

COVID-19: Corona Virus Disease 2019
SARS-CoV-2: severe acute respiratory syndrome coronavirus 2
fNIRS: functional near-infrared spectroscopy
HbO2: oxygenated hemoglobin
HbR: deoxygenated hemoglobin
PFC: prefrontal cortex
LS: lower limb Supplementary motor area
M1: primary motor cortex
ROIs: regions of interest
WHO: World Health Organization
fMRI: functional magnetic resonance imaging
IPAQ: International Physical Activity Questionnaire
FDR: false discovery rate
DMN: default mode network
*n*: Numbers
h: hours
*STD*: standard deviation
FC: functional connectivity
CH: Channel
S: source
D: detection
BMI: Body mass index
SPSS: Statistical package for social sciences
d: day
w: week
mm: millimeter
nm: nanometer
min: minutes

## 1 Introduction

Corona Virus Disease 2019 (COVID-19) is an infectious disease caused by severe acute respiratory syndrome coronavirus 2 (SARS-CoV-2) infection. After the COVID-19 pandemic ended, the prevalence of sedentary behavior among college students and its adverse effects on individual health have become public health issues of international concern.^1^According to the “2020 World Health Organization (WHO) Guidelines on Exercise and Sedentary Behavior",^2^ sedentary behavior increases the risk of physical and mental diseases for adults, including hypertension, cancer, and type 2 diabetes. Moreover, it affects mental health,^3^ cognitive health and sleep, as well as increases obesity, all-cause mortality and cardiovascular disease mortality, thus resulting in adverse outcomes.^4^, ^5^, ^6^ Additionally, it has a negative impact on students’ cognitive function and academic level.^7^ Due to their special study habits^8^ and lifestyle, college students have become are a large group with sedentary behavior, and as time passes, sedentary behavior among college students becomes increasingly serious.^9^ Furthermore, many studies have confirmed that^10^^,^^11^ one of the sequelae of COVID-19 infection is cognitive impairment,^12^ and a related change in brain functional connections at 6–9 months after recovery from COVID-19 has also confirmed the impact of COVID-19 on brain function.^13^

Intrinsic spontaneous neural activity occurs in the brain at rest, and functional connectivity refers to the time-series correlation or statistical dependence of neural activity between different regions of the brain.^14^ Functional near-infrared spectroscopy (fNIRS) is a noninvasive, nonionizing functional monitoring and imaging method for cerebral hemodynamics that is used to study healthy human brain function and various pathologies.^15^ It can indirectly reflect brain neural activity by detecting the contents of oxyhemoglobin (HbO_2_) and deoxyhemoglobin (reduced hemoglobin, or HbR) in the cerebral cortex in real time.^16^ Moreover, there is a good correlation between fNIRS and functional magnetic resonance imaging (fMRI).^17^ Due to its safety, quietness, resistance to motion artifacts and portability, fNIRS has become a popular method in brain research, as well as a promising research tool.^18^ As an important research group, college students usually need to sit for long periods of time during study, research and social interaction.^9^ Therefore, an understanding of the impact of prolonged sitting on their brain function can help us to focus more attention on their health and learning status. These findings can provide a basis for formulating more effective health intervention measures that are conducive to preventing and improving health problems related to sedentary behavior. Currently, there are no reports on the impact of sedentary behavior on the resting-state functional connectivity characteristics of college students after COVID-19 infection.

fNIRS was used to explore the resting brain functional connection characteristics of sedentary college students after recovering from COVID-19 and analyzed the impact of sedentary behavior and exercise on the functional connections of the prefrontal lobe, motor cortex and other brain areas of college students. To prevent and improve the relationship between college students and sedentary students, this study provides a reference on sitting-related health issues and promotes a healthy lifestyle and learning environment.

## 2 Materials and methods

### 2.1 Participants

This study included 44 sedentary college students from Harbin Sport University. Among them, there were 22 patients in the sedentary group (9 males and 13 females; age: [24.23 ± 1.72] years) and 22 patients in the exercise group (11 males and 11 females; age: $\left[24.09\;\pm \;1.97\right]$ years). All of the participants were right-handed, age-matched, and sex-matched. The baseline data characteristics of the patients are shown in Table 1.

**Table 1:** Age, anthropometric characteristics of participants and sedentary time.

Group	Sedentary Group (*n* ​= ​22)	Exercise Group (*n* ​= ​22)	*p*
Age (years)	24.23 ±1.72	24.09 ±1.97	0.814
Gender (male/female)	9/13	11/11	–
Height (cm)	169.23 ± 9.64	172.45 ± 9.16	0.316
Weight (kg)	66.27 ± 12.42	66.14 ± 9.32	0.969
BMI (kg·m^−2^)	22.93 ± 2.06	22.12 ± 1.22	0.118
Sedentary time (h)	7.07 ±0.88	6.80 ± 0.70	0.117

The values are expressed as the means ​± ​standard errors (*SE*).

BMI = Body mass index; h ​= ​hours; *n* ​= ​number.

The inclusion criteria were as follows.

(1) The experimental subjects must be college students.
(2) All of the subjects had a history of COVID-19 infection.
(3) The Physical Activity Readiness Questionnaire was completed to exclude subjects who could not participate in sports activities or who were suffering from sports diseases.
(4) The simplified version of the International Physical Activity Questionnaire was completed, and college students with sedentary behavior (sitting time ≥ 6 ​hour [h]/day [d]) were selected.
(5) Full participation in the exercise experiment was ensured.

The exclusion criteria included the following conditions.

(1) Infection or damage to the skin of the head.
(2) Communication or attention deficits that can interfere with experimental participation.
(3) Severe physical illness, history of brain trauma, neurological disease, drug or alcohol dependence, or other diseases that may affect brain structure and function.

Grouping basis. All of the subjects were invited to complete the Chinese version of the International Physical Activity Questionnaire (IPAQ) short version and sedentary behavior.^1^ The participants were divided into the following groups: (1) sedentary group, with a sitting time ≥ 6 ​h/d, an exercise frequency ≤ 3 times/week, and an exercise time ≤ 30 ​minute (min) (except for daily activities); and (2) exercise group, with a sitting time ≥ 6 ​h/d, an exercise frequency ≥ 4 times/week, a duration of exercise (including running, swimming, stair climbing, and fitness, among other activities) ​≥ ​5 ​h/week, a duration of each exercise ≥ 30 ​min, and a heart rate range of (220 - age) × (60%–80%).

### 2.2 Ethical approval

In this study, all of the participants were informed verbally and in writing about the purpose, nature, and procedures of the study. The experimental protocol was approved by the Ethics Committee of Harbin Sport University (2024001). Our study was conducted in accordance with the principles of the Declaration of Helsinki. All of the subjects were informed of the experimental procedures and signed written informed consent one day before testing.

### 2.3 Experimental protocol and data acquisition

Before the experiment was conducted, all of the participants received a comprehensive explanation of the experimental instructions. To minimize the interference of biological rhythms and dietary factors (such as irritating foods and drugs, including tobacco, alcohol, tea, and coffee, among other substances) on the experimental results, the subjects needed to fast for 12 ​h before the measurements and napped the night before. The participants stopped eating after 20:00 and 4:00. In addition, the subjects were asked to fall asleep before 23:00 the night before the test to ensure adequate rest. The experiment was initiated at 8:00 the next day and was conducted in a quiet environment, with only the tester and participants present during the test. To ensure data consistency, all of the tests were conducted by the same tester. The subjects sat quietly in a comfortable seat for 5 ​min before donning the headgear. During the scanning procedure, the subject was asked to close his or her eyes and remain relaxed but was also asked to stay awake and not fall asleep. Resting-state data collection lasted for 8 ​min.

**Fig. 1:**
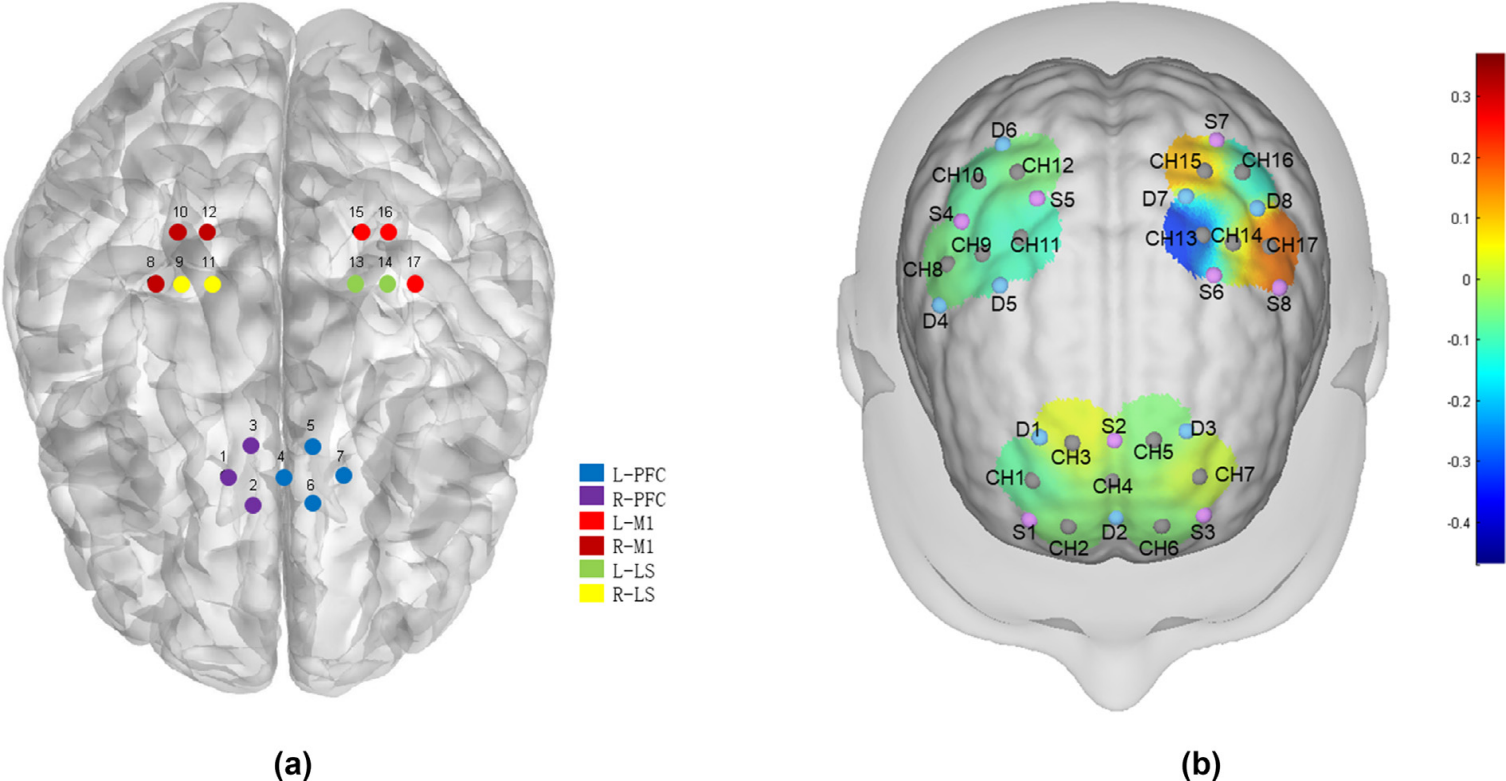
fNIRS signal acquisition diagram a: Correspondence diagram between the fNIRS acquisition head cap and the area of interest. b: fNIRS channel distribution. fNIRS: functional near-infrared spectroscopy.L-PFC: left prefrontal lobe; R-PFC: right prefrontal lobe; L-M1: left first somatic motor area; R-M1: right first somatic motor area; L-LS: left lower limb Supplementary motor area; R-LS: right lower limb Supplementary motor area. Pink circle = light source; blue circle = detector; gray circle = channel. A total of 8 emitting light sources (S) and 8 receivers (D) form a total of 17 channels (CH), covering the prefrontal lobes and motor areas on both sides. During the measurement process, A stronger signal corresponded to a redder color of the graph represented; a lower signal corresponded to a bluer color of the graph.

The 24-channel fNIRS imaging device NirSmart-3000A equipment (Danyang Huichuang Medical Equipment Co., Ltd., China) was used for the measurements. The equipment contains 8 light source probes and 8 detection probes, which constitute 17 effective channels in the experimental design. The distance between the headgear probe and the light source was 30 ​mm, and the brain area was below the midpoint of the channel (i.e., the midpoint of the connection between the emitter and the detector). The main detection area of this channel was positioned with reference to the international 10–20 system, covering the forehead and both sides of the movement area. Near-infrared light signals at two wavelengths (730 ​nm and 850 ​nm) were continuously recorded, with a sampling frequency of 11 ​Hz. The channel brain area was calibrated with reference to the Brodmann cerebral cortex partition. According to the coordinate information, the 17 channels were divided into 3 regions of interest (ROIs) in the left and right cerebral cortexes of the subject, including the right prefrontal cortex (R-PFC, CH1, 2, 3), left prefrontal cortex (L–PFC, CH4, 5, 6, 7), right primary motor cortex (R-M1, CH8, 10, 12), left primary motor cortex (L-M1, CH15, 16, 17), right lower limb Supplementary motor area (R-LS, CH9, 10), and left lower limb Supplementary motor area (L-LS, CH13, 14); additionally, the left and right sides were distinguished (Fig. 1), among which the lower limb Supplementary motor area (LS) represents a cortical brain functional area related to lower limb movement.

### 2.4 Data preprocessing

The NirSpark analysis system was used to analyze the time series concentrations of oxyhemoglobin (HbO_2_) and deoxyhemoglobin (HbR) in the 8-min resting-state data. Motion artifacts were eliminated, the original light intensity signal was converted into an optical density curve, a bandpass filter was used to eliminate physiological fluctuation noise (such as pulse and respiration, as well as baseline drift caused by environmental and temperature changes), and the modified Beer‒Lambert law was used to^19^ convert the optical density data into the concentrations of oxyhemoglobin (HbO_2_) and deoxygenated hemoglobin (HbR). The network module of the NirSpark software was used to extract the changes in HbO_2_ concentration at each time point, and the Pearson correlation coefficient of the HbO_2_ concentration in each channel in the time series was analyzed. After Fisher-*Z* transformation, the coefficient was defined as the functional connection strength between channels.

### 2.5 Statistical analysis

Statistical analysis was performed by using SPSS 27.0 software. The functional connection strength was tested to conform to the normal distribution and had homogeneous variances, represented by $\bar{\chi }\;\pm \;STD\;\left(\text{standard}\;\text{deviation}\right)$. The functional connection strength between the sedentary group and the exercise group was compared by using two independent samples *t* tests. The significance level was α ​= ​0.05, and false discovery rate (FDR) correction was performed.

## 3 Results

### 3.1 Brain functional connection characteristics and differences between the sedentary group and the exercise group based on HbO_2_

**Fig. 2:**
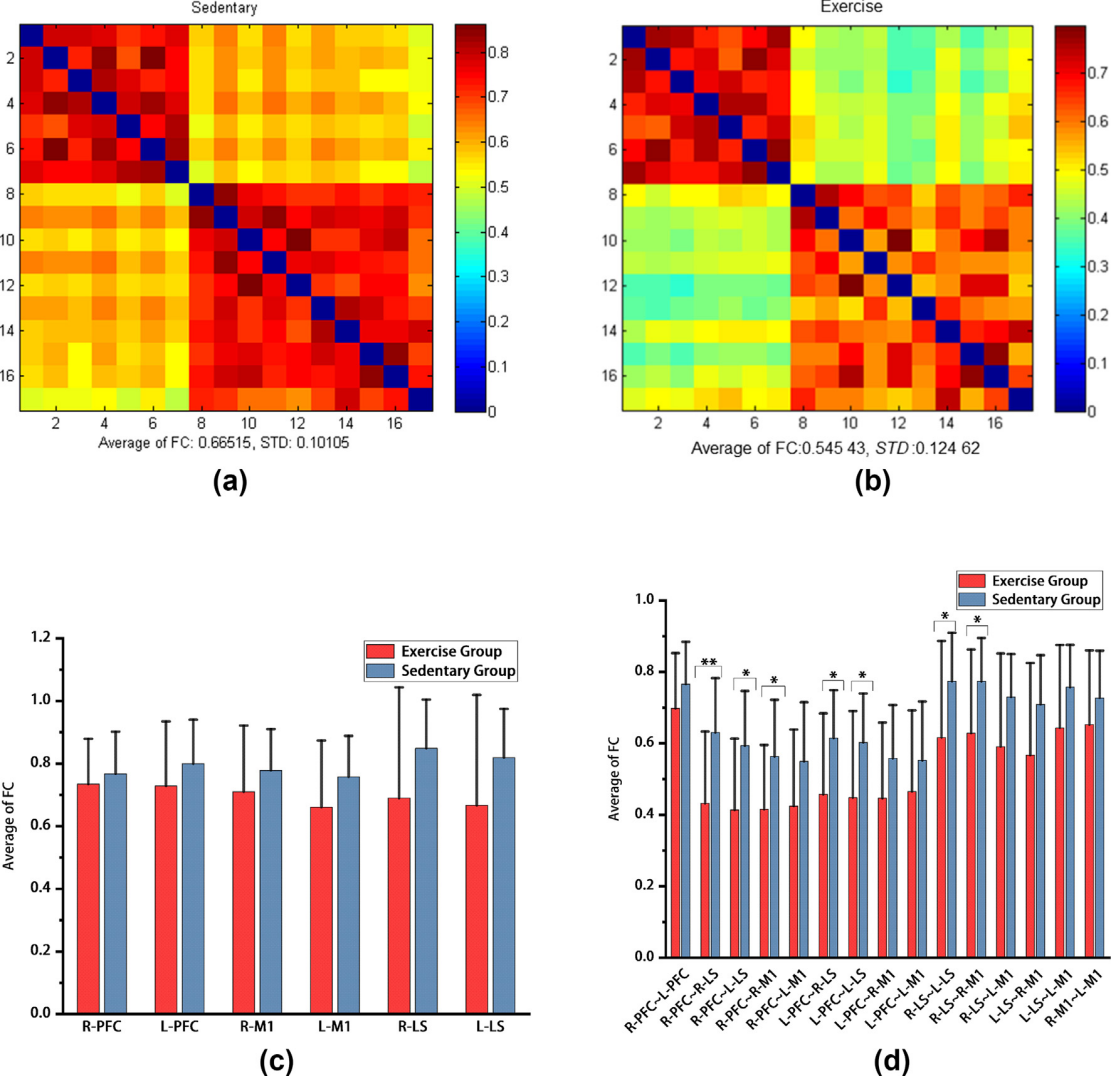
Average of functional connection strength based on HbO_2_ ​in the sedentary group and exercise group **a:** ​The average of functional connection strength based on HbO_2_ ​in the exercise group. b: The average of functional connection strength based on HbO_2_ ​in the exercise group. c: The mean difference in the average of functional connection strength of homologous ROIs based on HbO_2_ between the sedentary group and the exercise group. d: The mean difference in the average of functional connection strength of allogeneic ROIs based on HbO_2_ ​between the sedentary group and the exercise group. HbO_2_: oxygenated hemoglobin. Average of FC: average of functional connectivity. ​*STD*: standard deviation. L-PFC: left prefrontal lobe; R-PFC: right prefrontal lobe; L-M1: left first somatic motor area; R-M1: right first somatic motor area; L-LS: left lower limb Supplementary motor area; R-LS: right lower limb Supplementary motor area. ∗: ​*p* < 0.05; ∗∗: ​*p* < 0.01.

After calculating the mean value of the functional connection strength of the corresponding brain area channels in the resting state of the two groups of subjects, it was found that the average strength of the resting-state brain functional connection based on HbO_2_ in the sedentary group was 0.67±0.10 (Fig. 2a); in the exercise group based on HbO_2_, the average strength of the resting-state brain functional connections was 0.55±0.12 (Fig. 2b). The average strength of the resting-state brain functional connections in the sedentary group was significantly greater than that in the exercise group.

In the comparison of the mean functional connection strength of homologous ROIs between the sedentary group and the exercise group, there was no significant difference in the mean functional strength of ROIs between the two groups (p>0.05) (Fig. 2c); however, in the two groups of heterologous comparisons of the mean values of ROIs of functional connection strength, it was found that compared with that in the exercise group, the functional connection strength between the right PFC and the right LS (p=0.0097), the left LS (p=0.0127), and the right M1 (p=0.0305) in the sedentary group was significantly greater. As shown in Fig. 2, Fig. 4, the functional connection strength between the left PFC and the right LS (p=0.0312) and the left LS (p=0.0370) significantly increased; moreover, the functional connection strength between the right LS and the right M1 (p=0.0370), and the left LS (p=0.0438) significantly increased (Fig. 2d).

### 3.2 Brain functional connection characteristics and differences between the sedentary group and the exercise group based on HbR

**Fig. 3:**
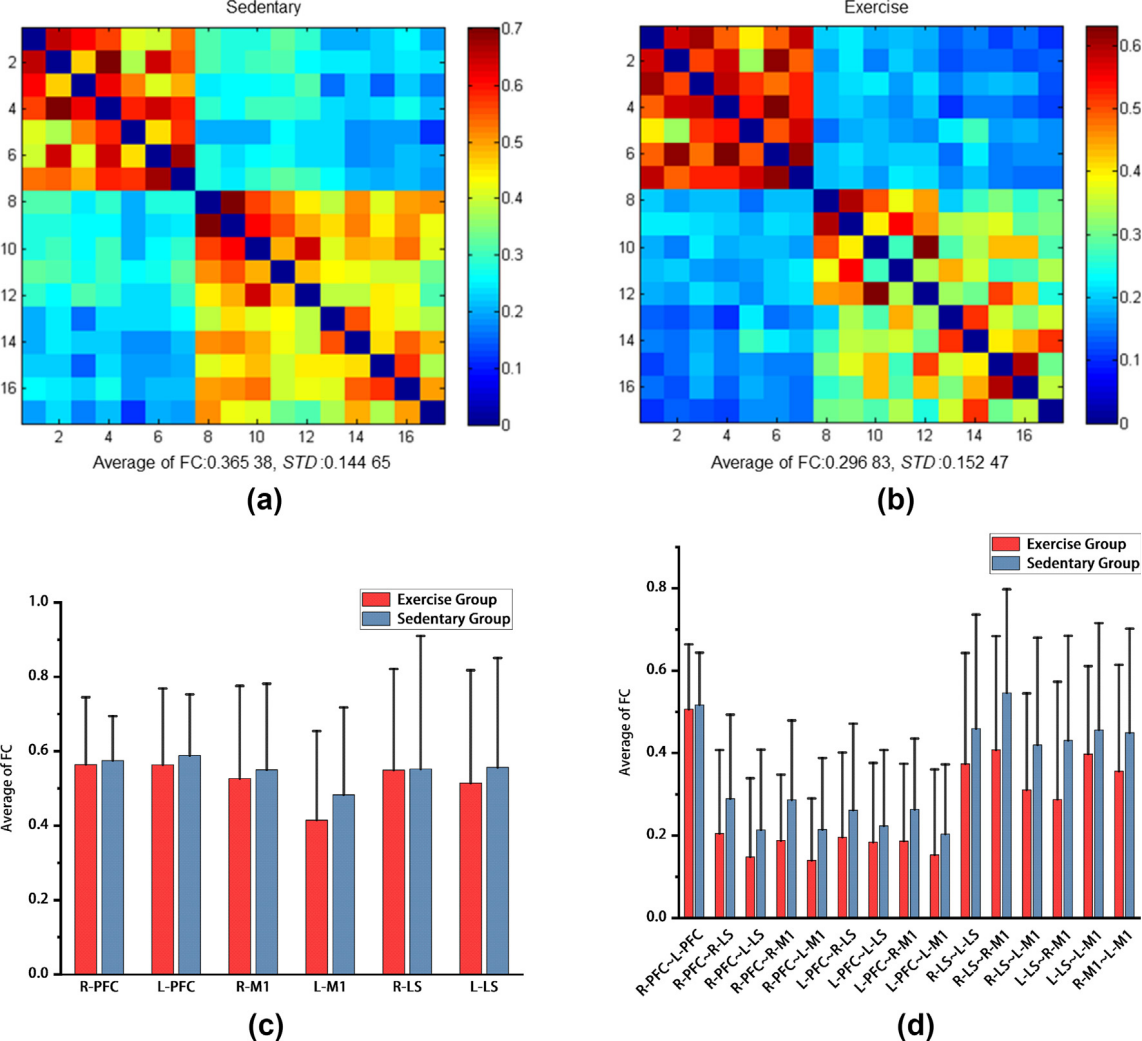
Average of functional connection strength based on HbR in the sedentary group and exercise group a: ​Average of functional connection strength based on HbR in the sedentary group. b: Average of functional connection strength based on HbR in the exercise group.HbR: deoxygenated hemoglobin. c: The mean difference in the average of functional connection strength of homologous ROIs based on HbR between the sedentary group and the exercise group. d: The mean difference in the average of functional connection strength of allogeneic ROIs based on the HbR between the sedentary group and the exercise group. HbR: deoxygenated hemoglobin. Average of FC: average of functional connectivity. *STD*: standard deviation. ROIs: regions of interest. L-PFC: left prefrontal lobe; R-PFC: right prefrontal lobe; L-M1: left first somatic motor area; R-M1: right first somatic motor area; L-LS: left lower limb Supplementary motor area; R-LS: right lower limb Supplementary motor area.

The resting-state brain functional connections of the two groups were calculated based on the HbR. The functional connection strength of the brain network in the sedentary group corresponding to ROIs was still greater than that in the exercise group. The average of functional connection strength of the two groups is shown in Fig. 3. The average strength of the resting-state brain functional connections based on the HbR in the sedentary group was 0.37±0.14 (Fig. 3a); moreover, the average strength of the resting-state brain functional connections based on the HbR in the exercise group was 0.37±0.15 (Fig. 3b).

**Fig. 4:**
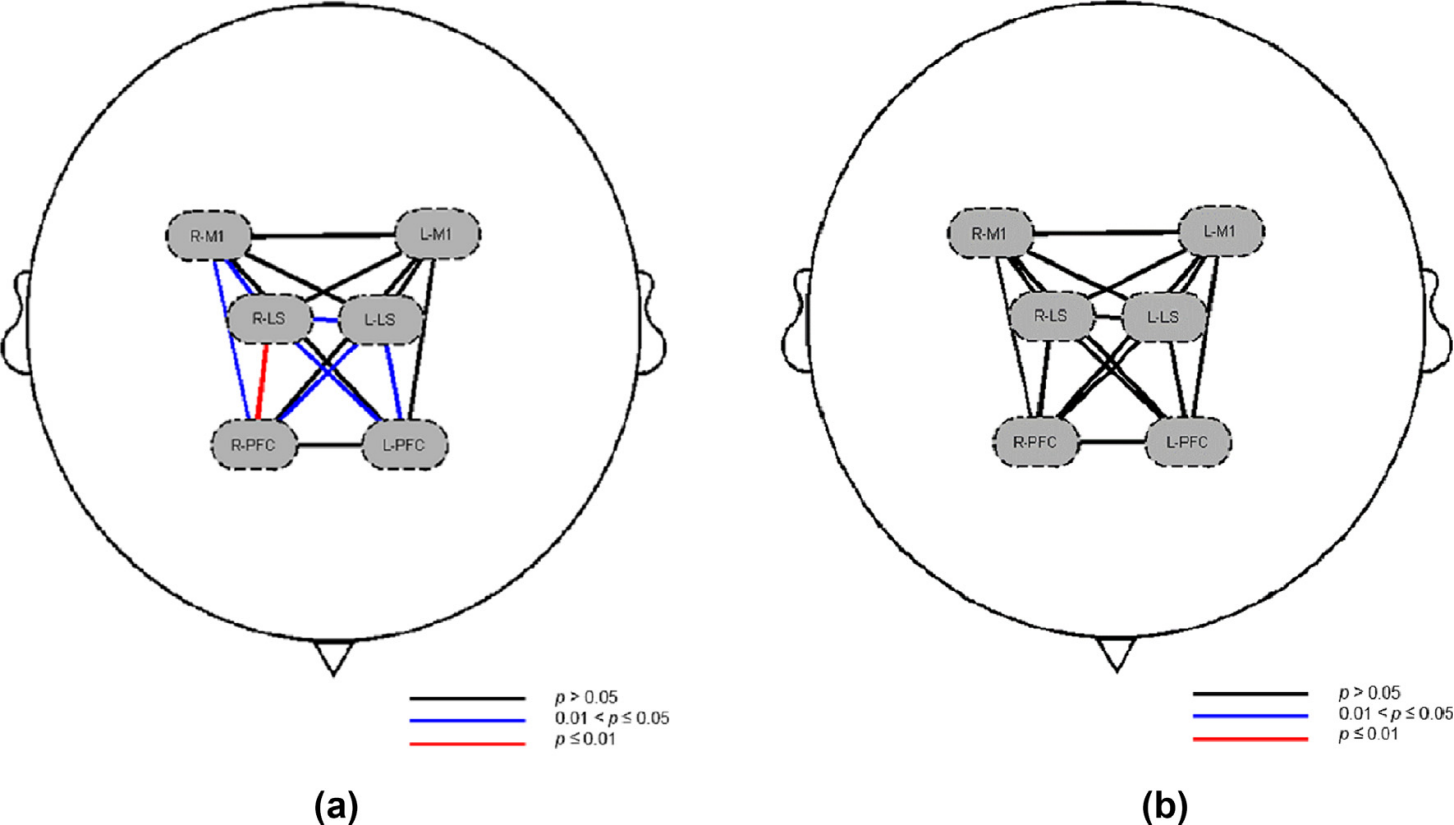
Differences in the average of functional connection strength of ROIs based on HbO_2_ ​and HbR between the sedentary group and the exercise group a: ​Differences in the average of functional connection strength of ROIs based on HbO_2_ between the sedentary group and the exercise group. b: Differences in the average of functional connection strength of ROIs based on HbR between the sedentary group and the exercise group. HbO_2_: oxygenated hemoglobin. HbR: deoxygenated hemoglobin. ROIs: regions of interest. L-PFC: left prefrontal lobe; R-PFC: right prefrontal lobe; L-M1: left first somatic motor area; R-M1: right first somatic motor area; L-LS: left lower limb Supplementary motor area; R-LS: right lower limb Supplementary motor area.

In the comparison of the mean functional connection strength of homologous ROIs based on HbR between the sedentary group and the exercise group, there was no significant difference in the mean functional strength of ROIs between the two groups (p>0.05) (Fig. 3c); similarly, in the long-term comparison of the mean functional connection strength of allogeneic ROIs based on HbR between the sitting group and the exercise group, there was no significant difference in the mean functional strength of ROIs between the two groups (p>0.05) (Fig. 3, Fig. 4).

## 4 Discussion

Long-term neuropsychiatric dysfunction is frequently observed after mild SARS-CoV-2 infection.^20^^,^^21^ Moreover, the COVID-19 pandemic has led to changes in most people's life behaviors, especially sedentary behavior^22^^,^^23^; however, there is a question as to whether this effect can persist. To determine the long-term impact of sedentary brain function on resting-state brain function, we selected sedentary college students as research subjects and used fNIRS technology to explore the effects of sedentary behavior on the resting-state cortical brain functional connection strength of college students after recovering from COVID-19. Analysis of the characteristics and differences in the resting-state functional connectivity of the cortical brain network between the sedentary group and the exercise group after recovery from COVID-19 demonstrated that the functional connectivity intensity of the sedentary group was greater than that of the exercise group, and this trend was obvious in the heterologous brain network.

### 4.1 fNIRS is an effective tool for studying the resting-state functional connections of cortical brain networks

Reports on resting-state functional connectivity related to sedentary behavior have mainly focused on functional magnetic resonance imaging (fMRI)-related studies.^24^ Compared with fMRI, fNIRS is inexpensive, easy to operate, has no noise interference, is easy to carry, has strong mobility, is less subject to site constraints, and is suitable for resting-state research in school environments. fNIRS uses two wavelengths (730 ​nm and 850 ​nm) to monitor changes in the concentrations of HbO_2_ and HbR in the cerebral cortex of subjects in real time. Theoretically, there is a strong correlation between HbR and HbO_2_, with a correlation coefficient close to −1. However, in practical applications, noise interferes with HbO_2_ and HbR data, and recording both variables at the same time can significantly improve the accuracy of brain-computer interfaces.^25^ Both the HbO_2_ and HbR signals have their own advantages and disadvantages. For example, HbO_2_ has a large change amplitude and high signal-to-noise ratio but is subject to more physiological interference; moreover, HbR is not easily affected by systematic changes but has low statistical power.^26^ Therefore, the future direction of research on sedentary behavior is to use fNIRS in multimodal mode to identify the impact of sedentary behavior on the resting-state functional connectivity of the whole brain at the HbO_2_ and HbR levels.

### 4.2 Prolonged sitting causes an increase in cortical brain network connection strength

In this study, the college students with sedentary behavior were in a time period of heavy academic workload. They sat for long periods of time every day to maintain their study progress. The results of this study are consistent with the results of an educational study on the promotion of cognitive function by sedentary behavior.^27^ We determined the following three speculations about the reasons explaining this phenomenon. (1) When college students engage in high-intensity learning while maintaining long-term sedentary behavior, the prefrontal lobe is highly activated and closely connected with other brain areas. However, this leads to sustained high activation of the default mode network (DMN) in the cerebral cortex during rest, potentially impacting brain function. fNIRS was used to study brain function by monitoring changes in the concentrations of HbO_2_ and HbR in the cerebral cortex of subjects. (2) People who sit for long periods of time also have a certain impact on cerebral blood vessels,^28^ but high-intensity learning tasks allow the body to adapt to the demand for blood supply to the brain. Many studies have shown that short-term, high-intensity acute exercise can improve the reduced blood supply to the brain caused by sedentary behavior,^29^^,^^30^ thus enhancing the brain's executive functions and improving cognition. (3) Furthermore, an fMRI-related study^13^ higher interconnectivity of the DMN in patients who reported of long-term symptoms after moderate SARS-CoV-2 infection, which is consistent with the findings of Zhang et al.^31^

This study demonstrated that sedentary behavior can enhance the strength of cortical brain network connections in the resting state of post-COVID-19 college students. However, whether excessive resting-state brain functional connection strength caused by long-term sedentary behavior is beneficial to the physical and mental health of college students still requires further exploration.

### 4.3 Moderate exercise can improve cortical brain network connection strength

Research has confirmed that different intensity exercise methods can improve brain function, especially because significant changes occur immediately after exercise.^32^^,^^33^ In the study of brain function in the resting state after long-term exercise, we found that the activation levels of the PFC, LS, and M1 in the right hemisphere of the sedentary group in the resting state were lower than those in the sedentary group. During the experiment, we ensured that both groups of subjects were sedentary; however, the exercise groups had good exercise habits (running, swimming, climbing stairs, and fitness, among other activities) and exercised for no less than 5 ​h per week. The heart rate is maintained at (220 - age) × (60%–80%) during exercise.^34^ Long-term exercise has a positive effect on brain oxygen supply^35^ and has a positive impact on cognitive and executive functions.^36^ An fNIRS study confirmed that^37^ functional connectivity is not improved as much as possible. Additionally, unilateral transcranial direct current stimulation led to a decrease in the activation state of the bilateral cerebral cortex. The researchers speculated that high-precision transcranial direct current stimulation can induce more effective interhemispheric neuronal signaling. In this study, the exercise group maintained good exercise habits despite sedentary behavior. The brain oxygen supply in the resting state can meet the oxygen demand of brain tissue at a lower flow rate. Moreover, after long-term exercise stimulation, the connection strength between the bilateral prefrontal lobes and exercise-related brain areas decreases, the connection efficiency between cortical brain areas increases, and the synchronization of brain areas increases.^38^

## 5 Conclusions

In this study, we used fNIRS to explore the effects of sedentary behavior on the resting-state cortical brain functional connection strength of college students after recovering from COVID-19. Furthermore, experimental results and graphical analysis based on functional connectivity showed that college student participants with sedentary behaviors had an increase in fNIRS signals while in a post-SARS-CoV-2 infection state and a sedentary context. This may indicate that sedentary behavior leads to an increase in the functional connectivity strength of the resting-state cortical brain network, particularly evidenced by significantly higher functional connectivity strength between the bilateral PFC, bilateral LS, and right M1 in the sedentary group. Meanwhile, the fNIRS signals decreased for the participants with exercise behaviors who maintained reasonable exercise routines under the same conditions as the sedentary participants. The outcome suggests that exercise behaviors can improve and reduce the impacts of sedentary behavior on the resting-state brain network, increase the connectivity efficiency between cortical brain networks, and enhance synchronicity across brain regions. This study provides new directions for future demonstrations of sedentary-related issues. Therefore, we encourage college students to use their favorite exercise methods to relax after a long period of sedentary behavior, which will be beneficial. Future research can also strive to measure the resting state of the whole brain area and use multimodal research methods to conduct a comprehensive and more in-depth exploration of the impact of sedentary behavior to better evaluate and understand the changes in neural mechanisms caused by sedentary behavior.

## 6 Limitations

First, this study did not detect infection of the brain tissue or peripheral nervous system of subjects after recovering from COVID-19, which is related to the uncontrollability of tissues related to SARS-CoV-2 infection. By conducting a COVID-19 questionnaire on the subjects, we determined that each subject had a history of at least one COVID-19 infection. Second, due to the fact that the exercise behaviors of the participants in the exercise group were not uniform (including running, swimming, climbing stairs, and fitness, among other activities), the results of the exercise group were uncontrollable. Therefore, to reduce this difference, we measure heart rate during the participant's exercise process and aim to maintain it at (220 - age) × (60%–80%). Third, the selection of subjects in this experiment was performed by completing subjective questionnaires, which are easily affected by the self-effect of the subjects. Future research can use more objective measurement methods (such as three-axis accelerometers)^39^ and multiangle research. Fourth, this study was conducted at the Harbin Sport University. The test population is limited and is susceptible to the potential influence of sample size and region. Therefore, the sample size and test area can be expanded in the future to explore the impact of sedentary behavior on people in different regions after recovering from COVID-19.

## Ethics approval statement

In this study, all of the participants were informed verbally and in writing about the purpose, nature, and procedures of the study. The experimental protocol was approved by the Ethics Committee of Harbin Sport University (2024001). Our study was conducted in accordance with the principles of the Declaration of Helsinki. All of the subjects were informed of the experimental procedures and signed written informed consent one day before testing.

## Submission statement

The manuscript has not been published and is not under consideration for publication elsewhere.

## Funding sources

This research was sponsored by the 2022 Heilongjiang Province Education and Teaching Reform Research General Project (Grant Number: SJGY20220644) and the Research Funding for Ph.D. Talent Introduction and Research Start-up Fees Project at Harbin Sport University (Grant Number: RC21-202206).

## Authors' contributions

**Xiaocong Yan:** Writing – original draft, Visualization, Software, Investigation, Formal analysis, Data curation. **Ying Qin:** Writing – original draft, Resources, Funding acquisition. **Haifeng Yu:** Formal analysis, Data curation. **Zhenghao Xue:** Supervision, Data curation. **Desheng Jiang:** Supervision, Data curation. **Limin Huang:** Writing – review & editing, Supervision, Conceptualization.

## Conflict of interest

The authors declare that they have no known competing financial interests or personal relationships that could have appeared to influence the work reported in this paper.
